# Improved adaptive impedance matching for RF front-end systems of wireless transceivers

**DOI:** 10.1038/s41598-020-71056-0

**Published:** 2020-08-21

**Authors:** Mohammad Alibakhshikenari, Bal S. Virdee, Pancham Shukla, Chan H. See, Raed A. Abd-Alhameed, Francisco Falcone, Ernesto Limiti

**Affiliations:** 1 Electronic Engineering Department, University of Rome “Tor Vergata”, Via del Politecnico 1, 00133 Rome, Italy; 2 Center for Communications Technology, School of Computing & Digital Media, London Metropolitan University, London, N7 8DB UK; 3 School of Engineering & the Built Environment, Edinburgh Napier University, Merchiston Campus, 10 Colinton Road, Edinburgh, EH10 5DT UK; 4 School of Engineering, University of Bolton, Deane Road, Bolton, BL3 5AB UK; 5 Faculty of Engineering and Informatics, University of Bradford, Bradford, BD7 1DP West Yorkshire UK; 6 Electric and Electronic Engineering Department, Universidad Pública de Navarra, Pamplona, Spain

**Keywords:** Electrical and electronic engineering, Electronics, photonics and device physics

## Abstract

In this paper an automatic adaptive antenna impedance tuning algorithm is presented that is based on quantum inspired genetic optimization technique. The proposed automatic quantum genetic algorithm (AQGA) is used to find the optimum solution for a low-pass passive *T*-impedance matching *LC*-network inserted between an RF transceiver and its antenna. Results of the AQGA tuning method are presented for applications across 1.4 to 5 GHz (satellite services, LTE networks, radar systems, and WiFi bands). Compared to existing genetic algorithm-based tuning techniques the proposed algorithm converges much faster to provide a solution. At 1.4, 2.3, 3.4, 4.0, and 5.0 GHz bands the proposed AQGA is on average 75%, 49.2%, 64.9%, 54.7%, and 52.5% faster than conventional genetic algorithms, respectively. The results reveal the proposed AQGA is feasible for real-time application in RF-front-end systems.

## Introduction

Antennas provide an interface with the propagating medium and therefore are essential components in wireless communication systems. There is a great demand for a single antenna that can operate efficiently over wide frequency band to accommodate software-defined multi-standard functionality^1^. Conventional wideband antennas for wireless systems are designed to perform satisfactorily, i.e. non-optimum, as the feed-point impedance cannot be matched properly over its operating band. Hence, at the transmitter the efficiency of the high power-amplifier is sub-optimum or at the receiver the low-noise amplifier performance is sub-optimum. To circumvent this issue an impedance matching network needs to be inserted between the antenna and transceiver. Impedance of antennas however can vary significantly with frequency as well as with operating conditions that can result in great impedance variations caused by various factors such as the proximity of a cellular phone to the user’s ear^2^. The use of a single impedance matching network with fixed *LC* component values is therefore unsuitable for realizing accurate matching over a wide frequency range. To realize optimum performance in terms of power and efficiency, it is therefore necessary to utilize an automatically tuneable impedance matching network that can dynamically control the antenna’s impedance, frequency band or operating conditions^3–21^. As yet automatically tuneable impedance matching networks are unsuitable for integration in wireless systems because tuning takes too long for practical applications.

Existing impedance tuning algorithms employ a step-by-step methodology where the passive tuning network is modified progressively until the required impedance match goal is realized^3,22^. Such gradient-based algorithms are computationally intensive because arrival at a solution involves calculation of matrix inversion that can often converge to localised optima^8,19^. Other algorithms investigated to date to circumvent such issues include (i) genetic algorithms (GA) that use non-gradient and global evolution optimising approaches^3,23^, and (ii) quantum genetic algorithms (QGA) that exploit the laws of quantum mechanics in order to perform efficient computation. Genetic algorithms are essentially adaptive search algorithms based on the evolutionary ideas by Charles Darwin of natural selection and genetics. These search algorithms operate on a set of elements, referred to as population, that evolves by means of crossover and mutation, towards a maximum of the fitness function. QGA have shown to be computationally efficient at solving problems like factorization^24^ or searching in an unstructured database^25^.

Taking advantage of the quantum to efficiently speed-up classical computation, the QGA outperforms the conventional genetic algorithms (CGA) when the fitness function is varying between genetic iterations. QGA updates the colony by combining quantum bit (qubit) coding with binary coding. It is a kind of efficient parallel algorithm. Qubits are exploited not only to represent the population, but also to perform fitness evaluation and selection. QGA uses the whole population at each genetic step, and in this sense, it can be considered to be a ‘global search’ algorithm.

Conventional genetic algorithms have been investigated for tuning the impedance of antennas^19,20,23^ but its use has been limited because of issues with obtaining an optimum goal and being slow. Although automatic genetic algorithm (AGA) is an attractive solution for software-defined transceivers it too faces the challenge of achieving an optimum solution quickly as the input impedance of the matching network is a non-linear function of load impedance. Therefore, a more efficient tuning algorithm is needed. Proposed in this paper is impedance tuning of antennas based on automatic quantum genetic algorithm (AQGA) that has been shown to be computationally efficient compared to CGA^25–30^ where the fitness function various continuously. The proposed AQGA is shown to arrive at an optimum impedance matching solution significantly quicker compared to conventional genetic algorithms. In fact, on average at 1.4 GHz band it is 75% faster,at 2.3 GHz band it is 49.2% faster,at 3.4 GHz band it is 64.9% faster; at 4.0 GHz band it is 54.7% faster; and at 5.0 GHz band it is 52.5% faster.

## Automatic antenna tuning system (AATS)

**Figure 1:**
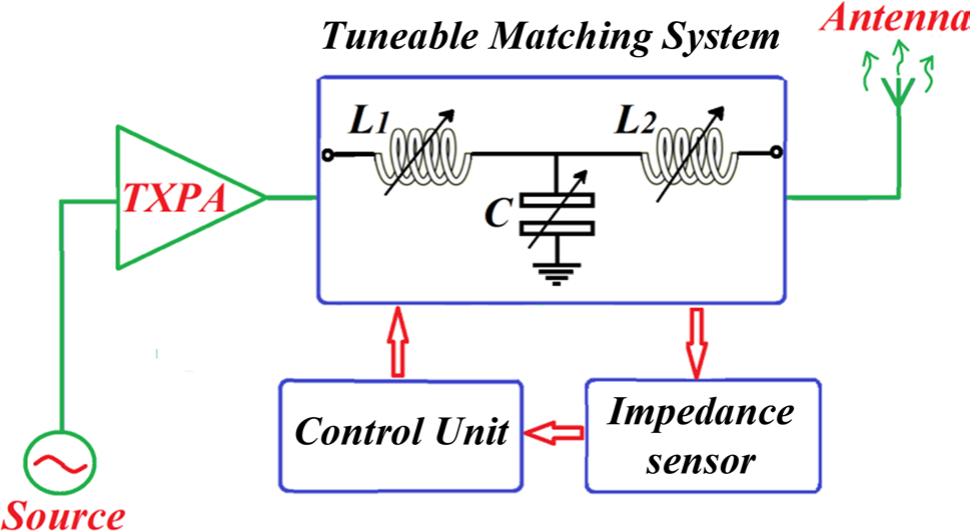
Block diagram of the automatic antenna tuning system (AATS).

The proposed automatic antenna tuning system is designed to conjugately match the impedance of the antenna with the transmitter’s impedance across the operating frequency band of the wireless system. The AATS, shown in Fig. 1, comprises (i) a tuneable matching network that generates the desired impedance transformation, (ii) an impedance sensor to measure the VSWR at the matching network input; and (iii) a control unit that modifies the impedance of the matching network to the desired impedance based on feedback from the impedance sensor by using the proposed tuning algorithm. The matching system can respond to variations in the antenna impedance affected by changes in its operating conditions.

## Impedance matching *LC*-network

As the antenna impedance is a function of the frequency and operational conditions, therefore the impedance matching network should be capable of being dynamically adaptable. The matching process involves suitably controlling the parameters of the matching network. The low-pass $\pi$-type and *T*-type impedance matching *LC*-networks are attractive configurations as they enable the use of a broad range of load impedances and harmonic-rejection characteristics^4,30^.

**Figure 2:**
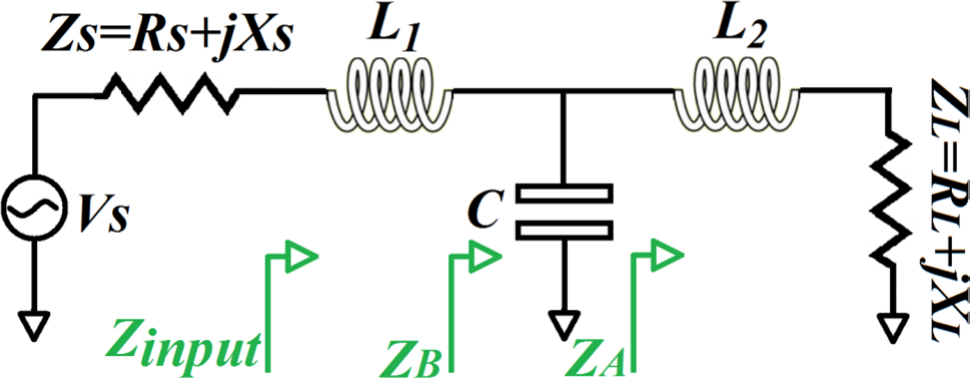
*T*-type impedance matching *LC*-network.

The proposed low-pass passive *T*-type impedance matching *LC*-network, which is located between the transmitter and antenna, is shown in Fig. 2, where $Z_{source}$ is the source impedance of the transmitter, and $Z_{Load}$ represents the load impedance. Impedances $Z_{A}$, $Z_{B}$ and $Z_{input}$ are annotated in Fig. 2 at each transformation stage from the load to the source. To realize impedance matching $Z_{input}$ must equal the real part of the source impedance, $R_{sousce}$. Hence, the maximum available power transfer to the load and Voltage Standing Wave Ratio (VSWR) can easily be determined from Fig. 2 as following

1 $$ Z_{A} = R_{Load} + j\left( {X_{Load} + \omega L_{2} } \right) $$

2 $$ Z_{B} = \frac{{R_{Load} + j\left[ {X_{A - } \omega C\left( {X_{A}^{2} + R_{Load}^{2} } \right)} \right]}}{{\left( {1 + \omega CX_{A} } \right)^{2} + \left( {\omega CR_{Load} } \right)^{2} }} $$

where $$X_{A} = X_{Load} + \omega L_{2}$$

3 $$ Z_{input} = Z_{B} + j\omega L_{1} $$

or

4 $$ Z_{input} = A + jB $$

where $$A = \frac{R}{{\left( {1 + \omega CX_{A} } \right)^{2} + \left( {\omega CR_{Load} } \right)^{2} }}$$ and $$B = \omega L_{1} + \frac{{X_{A - } \omega C\left( {X_{A}^{2} + R_{Load}^{2} } \right)}}{{\left( {1 + \omega CX_{A} } \right)^{2} + \left( {\omega CR_{Load} } \right)^{2} }}$$.

Reflection-coefficient ($\Gamma_{input}$) at input of the *LC*-network is defined by

5 $$ \Gamma_{input} = \frac{{Z_{input} - Z_{source} }}{{Z_{input} + Z_{source} }} $$

and

6 $$ VSWR = \frac{{1 + \left| {\Gamma_{input} } \right|}}{{1 - \left| {\Gamma_{input} } \right|}} $$

Maximum available power from source is

7 $$ P_{{max_{av} }} = \frac{{\left| {V_{source} } \right|^{2} }}{{4Re\left( {Z_{source} } \right)}} $$

Power delivered to the *LC*-network can be derived and is given by

8 $$ P_{input} = \frac{{\left| {V_{source} } \right|^{2} }}{{8Z_{o} }}\frac{{\left| {1 - {\Gamma }_{source} } \right|^{2} }}{{\left| {1 - {\Gamma }_{source} {\Gamma }_{input} } \right|^{2} }}\left( {1 - \left| {{\Gamma }_{input} } \right|^{2} } \right) $$

where $\Gamma_{soarce}$ is reflection-coefficient at the source, and $Z_{o}$ is the characteristic impedance.

**Figure 3:**
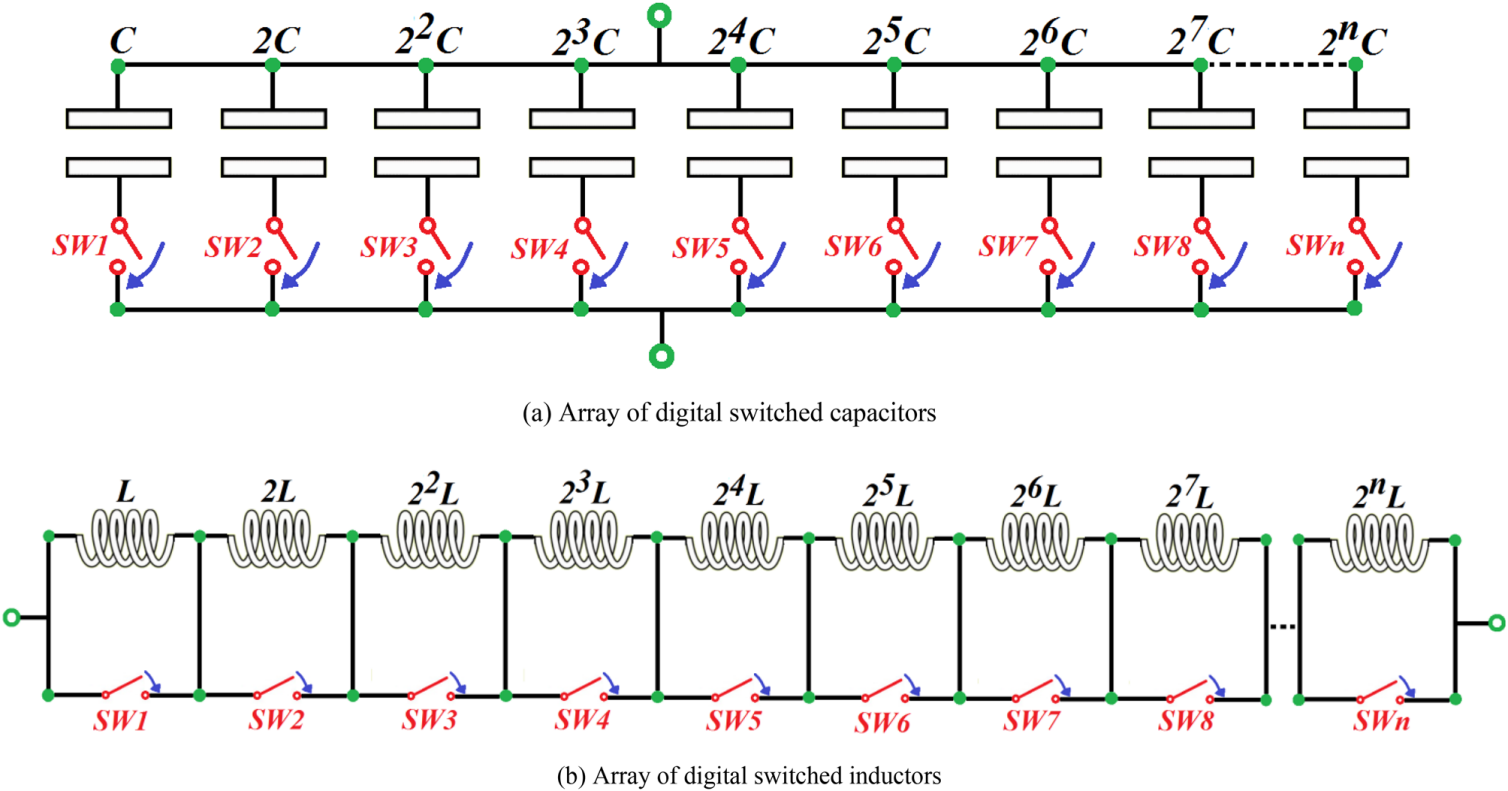
Digital switched *LC*-network arrays.

In the proposed *T*-type impedance matching *LC*-network the magnitude and range of the reactive components should cover the impedances necessary to match the antenna across the wireless systems operating bandwidth. The reactive components should be controllable to accommodate variations in the antenna’s operating conditions. To implement the tuning requirement an array of digital switches of fixed value reactive component are deployed, as shown in Fig. 3. This arrangement facilitates automatic adjustment of the matching network to meet the matching condition (ideally $\Gamma_{source} = 0$ and $VSWR = 1$) using a tuning algorithm.

## Proposed automatic quantum genetic algorithm

Fast tuning algorithm is required for antenna impedance networks especially with changing loads and operational conditions. In the case where many network combinations are possible it is important to have a tuning algorithm that can reduce the number of networks possible. This can be achieved by generating a look-up table of matching networks for various frequencies in the operating range. This approach will facilitate the control system to rapidly select a suitable tuning network as a function of frequency^8^. The disadvantage of this approach is it’s not able to respond quickly to changes in the antenna’s impedance as a function of time without repeating the whole process again.

Genetic tuning algorithms, however, based on iterations converge on the best impedance networks^23,25,26^. Such algorithms must initially perform many iterations to arrive at a satisfactory impedance solution with no need for explicit rules. But with continued operation such algorithms achieve matching with fewer iterations. The disadvantage of GA is it requires significant computational time.

It has been shown that AQGA is an efficient algorithm^23,27–30^ as it’s (i) less prone to being trapped in a localised optimum solution,(ii) requiring less iteration; and (iii) it converges more rapidly to a final solution. It is for these reasons the tuning mechanism chosen here is based on AQGA.

### Representation

AQGA is a quantum inspired genetic algorithm that is based on quantum computation and conventional GA. Its states are a superposition of qubits^23,27^. A qubit can assume state |0 > or |1 > , or any superposition of the dual states defined by |$$\delta$$ >  = $$ i$$|0 >  + $$j$$|1 > , where parameters $$i$$ and $$j$$ represent complex numbers indicating the probability-amplitudes of the respective states. For a system of *n* qubits, the system can exhibit $$2^{n}$$ states concurrently. Its representation is given by ^4,30^

9 $$ \left[ {\left. {\begin{array}{*{20}c} {i_{1} } \\ {j_{1} } \\ \end{array} } \right|\left. {\begin{array}{*{20}c} {i_{2} } \\ {j_{2} } \\ \end{array} } \right|\left. {\begin{array}{*{20}c} {i_{3} } \\ {j_{3} } \\ \end{array} } \right|\left. {\begin{array}{*{20}c} {i_{4} } \\ {j_{4} } \\ \end{array} } \right|\left. {\begin{array}{*{20}c} {i_{5} } \\ {j_{5} } \\ \end{array} } \right|\left. {\begin{array}{*{20}c} \ldots \\ \ldots \\ \end{array} } \right|\begin{array}{*{20}c} {i_{n} } \\ {j_{n} } \\ \end{array} } \right] $$

where

10 $$ \left| {i_{x} } \right|^{2} + \left| {j_{x} } \right|^{2} = 1,\quad x = 1, 2, 3, 4, 5, \ldots ., n $$

One qubit chromosome in Eq. (9) can represent all possible states in the primary stages of evolution, whereas 2^*n*^ chromosomes are required in a classical system.

For the *T*-type impedance matching *LC*-network in Fig. 2, the components ($L_{1}$, $L_{2}$, and $C$) are coded in the form of Eq. (9). The chromosome $\left[ {L_{1} ,L_{2} ,C} \right]$ comprises 30-bit qubits, where 10-bit qubits represent each component. The quantum algorithm’s challenge is to determine the minimum list of *M* items.

### Structure of AQGA

The structure of the AQGA for modifying the *T*-type impedance matching *LC*-network is thus^4,30^:

(i) *Initialize the binary instants and the quantum population (do this for each generation)*

(ii) *Compute the fitness of each entity* $P_{Y}^{x}$

(iii) *Categorize the entities related to the fitness amounts and store the best chromosome*

(iv) *Apply the quantum genetic operators on* $P_{Quantum}^{x}$*and update qubit chromosome applying rotation matrix*

The initial component magnitudes of the *T*-type impedance matching *LC*-network are achieved from the previous section and are adjusted by random numbers where the magnitudes of the probability amplitudes are selected in a random fashion from the intervals of $L_{1} , L_{2} \in \left\{ {10^{ - 12} ,10^{ - 6} } \right\}$ and $C \in \left\{ {10^{ - 14} ,10^{ - 7} } \right\}$. Constituents of the quantum population are represented as

11 $$ P_{Quantum}^{x} = \left[ {Q_{1}^{x} , Q_{2}^{x} , Q_{3}^{x} , Q_{4}^{x} , Q_{5}^{x} , \ldots , Q_{m}^{x} } \right] $$

where

12 $$ Q_{y}^{x} = \left\{ {i_{y,l}^{x} ,j_{y,l}^{x} } \right\}^{R} $$

$Q_{y}^{x}$ is the $l{th}$ qubit size in the $x{\rm th}$ generation of the $y{\rm th}$ constituent in the quantum population ($P_{Y}^{x}$) and $m$ is the population size. $P_{Y}^{x}$ is generated from qubit chromosome $P_{Quantum}^{x}$, and represented as

13 $$ P_{Y}^{x} = \left[ {Y_{1}^{x} , Y_{2}^{x} , Y_{3}^{x} , Y_{4}^{x} , Y_{5}^{x} , \ldots , Y_{m}^{x} } \right] $$

where $Y_{y}^{x}$ is the $x{\rm th}$ bit of the chromosome.

The quantum matrix is transformed into a binary matrix in the measurement operation. As done in other quantum systems a single solution is extracted from the quantum matrix while preserving all other configurations. The magnitude of the qubit is determined according to its probability pairs $\left| {i_{y,l}^{x} } \right|^{2}$ and $\left| {j_{y,l}^{x} } \right|^{2}$. Population of binary entities is built from the quantum population $P_{Quantum}^{x}$. Each qubit is observed for any destruction in the qubit chromosome. Diversity in population is achieved by generating a random number. |1 > state is recorded whenever the magnitude of the random number is bigger than the corresponding chromosome probability amplitude. If the magnitude of the random number is smaller compared to the corresponding chromosome probability amplitude |0 > state will be observed.

In the evaluation phase the fitness of the current and the best chromosome is given by $f\left( {CH_{\beta }^{\alpha } } \right)$ and $f\left( {CH_{best} } \right)$, respectively, where $CH_{best}$ is the $\alpha{\rm th}$ bit of the best chromosome. $f\left( {CH_{best} } \right)$ is obtained from

14 $$ f\left( {CH_{best} } \right) = maximum\left\{ {f\left( {CH_{1}^{\alpha } } \right), f\left( {CH_{2}^{\alpha } } \right), f\left( {CH_{3}^{\alpha } } \right), f\left( {CH_{4}^{\alpha } } \right), f\left( {CH_{5}^{\alpha } } \right), \ldots , f\left( {CH_{m}^{\alpha } } \right)} \right\} $$

For the *T*-type impedance matching *LC*-network, the population is evaluated by

15 $$ f\left( {L_{1} ,L_{2} ,C} \right) = \left( {1 + \left| {Z_{source} - Z_{input} } \right|^{2} } \right)/\left| {\Gamma_{source} } \right| $$

where $Z_{input}$ is the population’s actual input impedance, and $\Gamma_{source}$ is the source’s actual reflection-coefficient. The aim of the tuning algorithm is to determine the values of $L_{1}$, $L_{2}$, and $C$ to meet the matching conditions. This is followed by updating the *xth* population of qubit chromosomes $P_{Quantum}^{x}$ by applying quantum rotation.

Qubit chromosome of $P_{Quantum}^{x}$ with the best fitness is chosen for each binary chromosome $P_{Y}^{x}$. Then qubit chromosomes are sorted according to the values of the fitness in every iteration executed. To avoid convergence to a local maximum, the selection strategy used here was to extract the optimum and part of "*not so good*" entities. In this way global optimisation is achieved. A uniform quantum crossover operation is applied thereafter to the chosen entities. This is followed by mutating the probability amplitude of each qubit chromosome to generate new entities. Finally, the quantum chromosome is updated by using quantum rotation. The rotation matrix is represented by

16 $$ R\left( \varphi \right) = \left[ {\begin{array}{*{20}c} {{\sin}\left( \varphi \right)} & {cos\left( \varphi \right)} \\ {cos\left( \varphi \right)} & { - sin\left( \varphi \right)} \\ \end{array} } \right] $$

where $\varphi$ represents the angle of the rotation. The $l{\rm th}$ qubit in the $x{\rm th}$ generation is

17 $$ Q_{y}^{x} = \left\{ {i_{y,l}^{x} ,j_{y,l}^{x} } \right\}^{R} $$

which is updated as

18 $$ \left[ {\begin{array}{*{20}c} {i_{y,l}^{x} } \\ {j_{y,l}^{x} } \\ \end{array} } \right]_{new} = T\left( {\varphi_{y,l}^{x} } \right)\left[ {\begin{array}{*{20}c} {i_{y,l}^{x} } \\ {j_{y,l}^{x} } \\ \end{array} } \right]_{old} $$

where $\varphi_{y,l}^{x}$ is the corresponding rotation angle of $Q_{y}^{x}$.

19 $$ Q_{y,new}^{x} = \left\{ {i_{y,l}^{x} ,j_{y,l}^{x} } \right\}_{new}^{R} $$

20 $$ Q_{y,old}^{x} = \left\{ {i_{y,l}^{x} ,j_{y,l}^{x} } \right\}_{old}^{R} $$

21 $$ \varphi_{y,l}^{x} = d_{y,l}^{x} \left| {\varphi_{y,l}^{x} } \right| $$

where $\left| {\varphi_{y,l}^{x} } \right|$ is its amplitude and $d_{y,l}^{x}$ is its sign. $d_{y,l}^{x}$ is dependent on Ψ = $i_{y,l}^{x}$. $j_{y,l}^{x}$. Table 1 shows the relation between the fitness condition $d_{y,l}^{x}$ and "Ψ", where the mark sensing Ψ > 0 stands *Re*(Ψ).*Im*(Ψ) > 0, and Ψ < 0 denotes for *Re*(Ψ).*Im*(Ψ) < 0. In case of Ψ = 0, i.e. $i_{y,l}^{x}$ = 0 or $j_{y,l}^{x}$ = 0, there are different $d_{y,l}^{x}$ amounts, whose details are presented in Table 1. The symbolism ‘*’ illustrates arbitrary state, symbol “1” in the columns demonstrates for the clockwise rotation of $d_{y,l}^{x}$, and “0” in the columns corresponded to $d_{y,l}^{x}$ indicates that rotation is not accomplished. The rotation operation helps AQGA to converge rapidly to find a solution.

**Table 1:** Strategy of the quantum rotation.

$CH_{\beta }^{\alpha }$	$CH_{best}$	$f\left( {CH_{\beta }^{\alpha } } \right)$ ≥ $f\left( {CH_{best} } \right)$	$\left| {\varphi_{y,l}^{x} } \right|$	$d_{y,l}^{x}$			
				Ψ > 0	Ψ < 0	Ψ = 0	
						$i_{y,l}^{x}$ = 0	$j_{y,l}^{x}$ = 0
1	1	*	*	0	0	0	0
1	0	*T (true)*	0.08*π*	− 1	1	*	0
1	0	*T*	0.04*π*	1	− 1	0	*
0	1	*F (false)*	0.08*π*	1	− 1	0	*
0	1	*F*	0.04*π*	− 1	1	*	0
0	0	*	*	0	0	0	0

## Results and discussions

**Figure 4:**
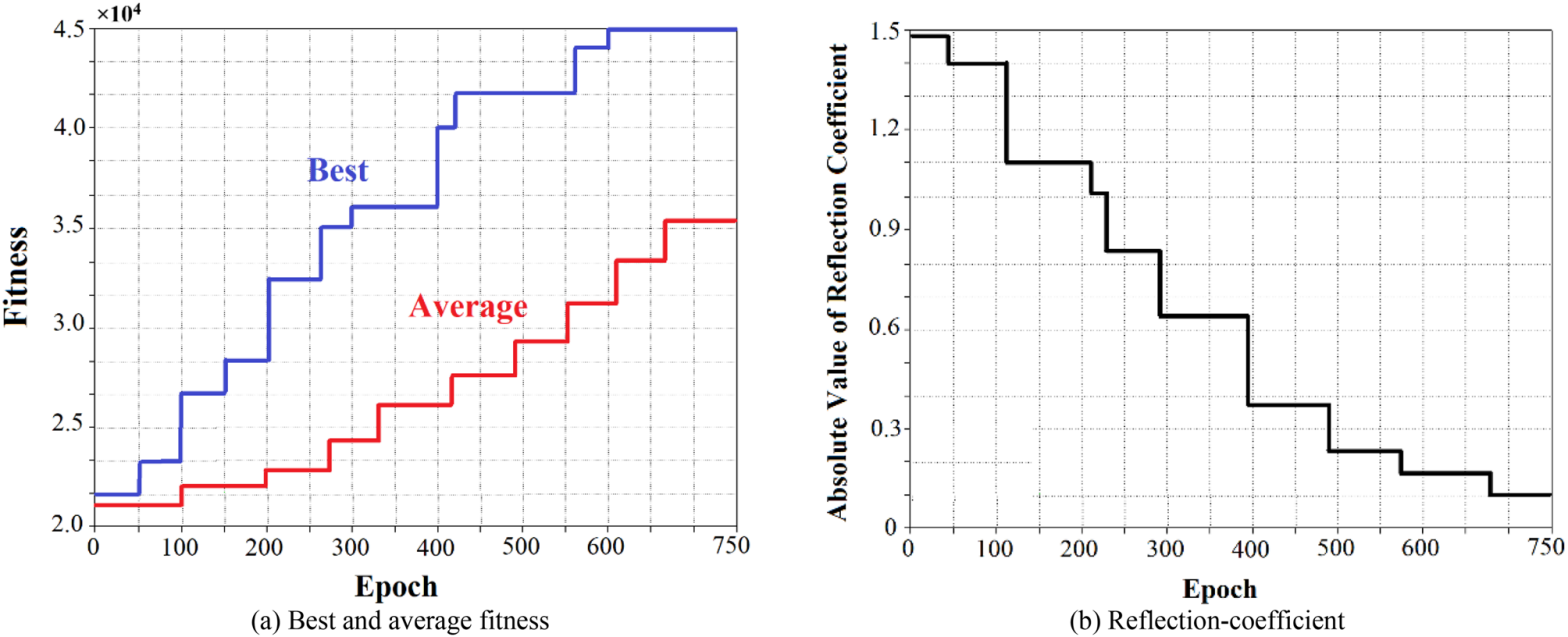
Training plots of the AQGA tuning for $Z_{source}$ = 50 + j30 Ω, $Z_{Load}$ = 75 + j50 Ω, signal frequency of 5 GHz (WiFi band), & signal amplitude of 1 V.

The proposed automatic quantum GA tuning approach is compared with the conventional GA methodology to verify its effectiveness. It was therefore necessary to extensively simulate both tuning approaches. The tuning times of the algorithms was assessed over 400 simulations to determine the algorithm’s speed in arriving at the optimal solution. The training diagrams of the AQGA tuning approach are shown in Fig. 4 for the following conditions: $Z_{source}$ = 50 + j30 Ω, $Z_{Load}$ = 75 + j50 Ω, signal frequency of 5 GHz (WiFi band), and signal amplitude of 1 V. The results shown are a function of size of genetic population, probability of mutation, cross-probability, specific mutation, cross and selection operations. The AQGA tuning was executed many times for various parameter magnitudes. The best and average fitness results in Fig. 4 corresponds to a population size of 100 entities, a mutation probability of 0.5% and a cross-probability of 12.4%. The best entities are chosen by the probability of 85% and the remainders are chosen by the probability of 15%. The algorithm terminated after 750 generations and the optimum solution achieved at this point is applied, which results in $L_{1}$ = 5.62 *nH*, $L_{2}$ = 3.45 *nH*, and $C$ = 2.18 *pF*. The quantum elapsed time is 7.25 s. Figure 4(b) shows the corresponding reflection-coefficient variations with training epochs. Table 2 also gives the different load impedance results at 5 GHz (WiFi band).

**Table 2:** Results of 400 runs for different load impedances at various frequencies using the proposed AQGA and CGA tuning approaches.

Frequency band		*Z*_*Load*_ (Ω)	Train epochs	Relative error (%)	*VSWR*
CGA	*f* = 1.4 GHz (1,350.5–1,515 MHz) (satellite services)	25 + j50	332,542	2.45 × 10^−4^	1.2492
		25 − j50	525,278	2.97 × 10^−5^	1.3548
		50 + j75	576,985	6.55 × 10^−3^	1.2254
		50 − j75	548,481	4.92 × 10^−5^	1.1458
		75 + j100	188,094	1.58 × 10^−4^	1.2094
		75 − j100	179,792	2.14 × 10^−3^	1.1983
AQGA	*f* = 1.4 GHz (1,350.5–1,515 MHz) (satellite services)	25 + j50	270	9.86 × 10^−6^	1.1368
		25 − j50	328	3.71 × 10^−6^	1.0557
		50 + j75	360	2.07 × 10^−3^	1.1521
		50 − j75	345	1.21 × 10^−4^	1.0692
		75 + j100	290	1.70 × 10^−5^	1.0958
		75 − j100	265	1.55 × 10^−4^	1.1410
CGA	*f* = 2.3 GHz (2,350–2,390 MHz) (LTE networks)	25 + j50	31,347	2.26 × 10^−3^	1.4059
		25 − j50	21,942	3.98 × 10^−3^	1.2602
		50 + j75	20,563	1.62 × 10^−3^	1.3058
		50 − j75	30,725	2.81 × 10^−3^	1.4069
		75 + j100	19,497	4.20 × 10^−4^	1.3693
		75 − j100	22,051	5.95 × 10^−4^	1.1690
AQGA	*f* = 2.3 GHz (2,350–2,390 MHz) (LTE networks)	25 + j50	256	6.83 × 10^−4^	1.2275
		25 − j50	291	3.12 × 10^−5^	1.1508
		50 + j75	275	1.03 × 10^−4^	1.1821
		50 − j75	213	3.55 × 10^−4^	1.2551
		75 + j100	272	4.07 × 10^−4^	1.1953
		75 − j100	219	6.73 × 10^−5^	1.0565
CGA	*f* = 3.4 GHz (3,410–3,600 MHz) (radar systems)	25 + j50	28,653	3.24 × 10^−3^	1.3429
		25 − j50	32,961	4.01 × 10^−2^	1.2630
		50 + j75	25,061	3.98 × 10^−4^	1.2918
		50 − j75	19,239	1.53 × 10^−3^	1.7392
		75 + j100	24,716	3.65 × 10^−3^	1.2530
		75 − j100	15,630	2.64 × 10^−4^	1.3709
AQGA	*f* = 3.4 GHz (3,410–3,600 MHz) (radar systems)	25 + j50	189	2.02 × 10^−4^	1.2093
		25 − j50	267	6.16 × 10^−3^	1.1196
		50 + j75	345	3.86 × 10^−3^	1.1532
		50 − j75	220	5.37 × 10^−4^	1.0514
		75 + j100	198	1.17 × 10^−3^	1.1090
		75 − j100	254	4.29 × 10^−5^	1.0961
CGA	*f* = 4.0 GHz (3,700–4,200 MHz) (satellite earth stations)	25 + j50	234,975	6.32 × 10^−3^	1.3095
		25 − j50	286,531	4.61 × 10^−2^	1.2783
		50 + j75	295,514	2.84 × 10^−4^	1.2590
		50 − j75	301,860	1.90 × 10^−3^	1.4061
		75 + j100	287,461	3.66 × 10^−3^	1.3068
		75 − j100	310,639	2.38 × 10^−4^	1.2831
AQGA	*f* = 4.0 GHz (3,700–4,200 MHz) (satellite earth stations)	25 + j50	429	7.51 × 10^−4^	1.1009
		25 − j50	401	1.39 × 10^−3^	1.1891
		50 + j75	385	1.01 × 10^−4^	1.2029
		50 − j75	393	4.42 × 10^−4^	1.3007
		75 + j100	438	2.58 × 10^−3^	1.2117
		75 − j100	419	5.98 × 10^−5^	1.1503
CGA	*f* = 5.0 GHz (5,350–5,925 MHz) (WiFi band)	25 + j50	28,537	2.47 × 10^−3^	1.4280
		25 − j50	19,620	3.85 × 10^−3^	1.3891
		50 + j75	15,352	1.29 × 10^−4^	1.3631
		50 − j75	22,385	5.38 × 10^−2^	1.3284
		75 + j100	29,716	4.50 × 10^−3^	1.2801
		75 − j100	18,630	2.48 × 10^−3^	1.4429
AQGA	*f* = 5.0 GHz (5,350–5,925 MHz) (WiFi band)	25 + j50	247	1.24 × 10^−4^	1.1503
		25 − j50	209	4.92 × 10^−4^	1.2745
		50 + j75	278	1.01 × 10^−5^	1.1645
		50 − j75	192	1.58 × 10^−2^	1.1716
		75 + j100	273	3.54 × 10^−4^	1.1550
		75 − j100	203	4.97 × 10^−4^	1.3509

**Figure 5:**
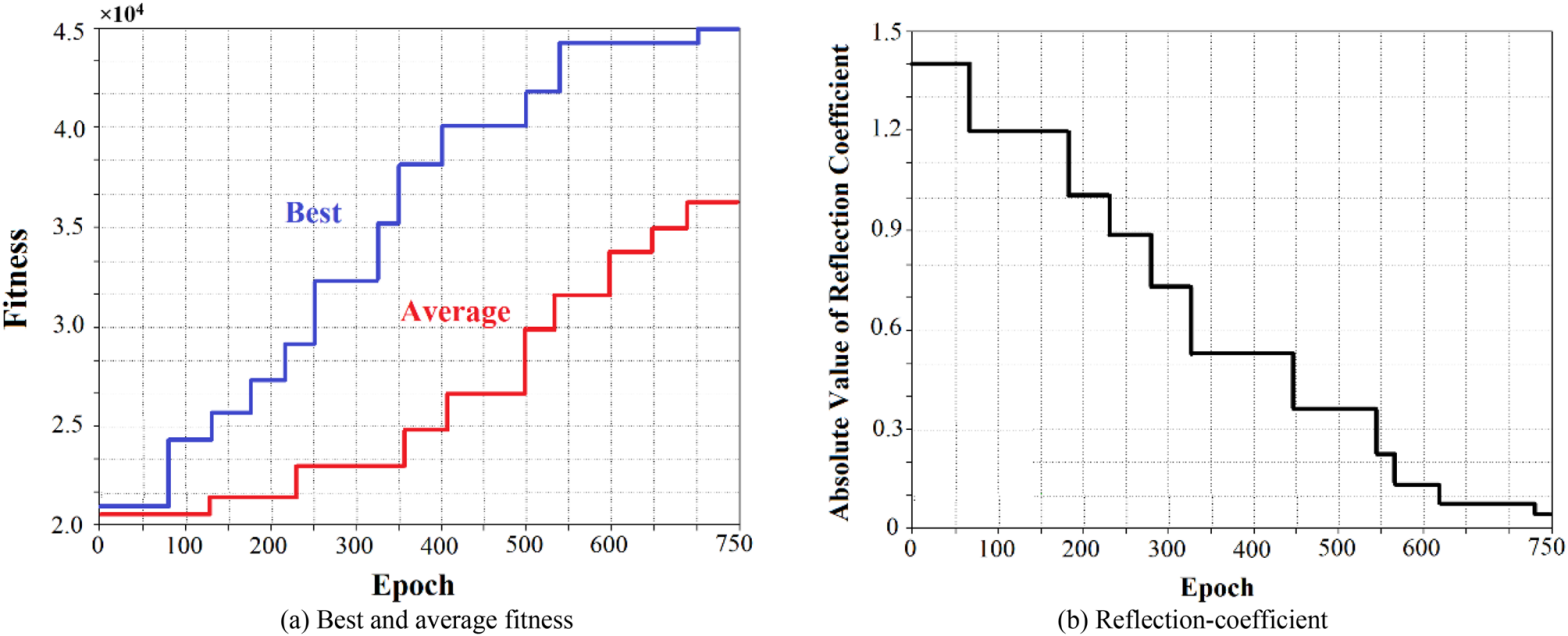
Training curves of the AQGA tuning for $Z_{source}$ = 50 + j30 Ω, $Z_{Load}$ = 75 + j50 Ω, signal frequency of 3.4 GHz (radar systems), & signal amplitude of 1 V.

In another example, AQGA tuning is applied to source impedance of $Z_{source}$ = 50 + j30 Ω, load impedance of $Z_{Load}$ = 75 + j50 Ω, source signal frequency of 3.4 GHz (radar systems), and signal amplitude of 1 V. Computational results in Fig. 5 are for a population size of 100 entities, a mutation probability of 0.6% and a cross-probability of 13.6%. Best entities are chosen by the probability of 87% and the remainders are selected by the probability of 13%. The algorithm terminates after 750 generations and the optimum goal achieved gives $L_{1}$ = 7.11 *nH*, $L_{2}$ = 4.25 *nH*, and $C$ = 3.05 *pF*. The quantum elapsed time is 7.02 s. Table 2 also gives the results for different load impedances at 3.4 GHz (radar systems).

Statistical results of 400 executions for various load impedances applying the proposed AQGA are presented in Table 2 for different frequency bands, i.e. 1.4 GHz (military and satellite services), 2.3 GHz (LTE networks), 3.4 GHz (radar systems), 4.0 GHz (satellite earth stations), and 5.0 GHz (WiFi band), where the relative error in input impedance is defined as

22 $$ E_{Relative} = \left| {Z_{input} - Z_{source} } \right|/Z_{source} $$

where $Z_{input}$ is the input impedance determined from the tuning algorithm, and $Z_{source }$ = 50 + j30 Ω is the source impedance to be matched. As can be seen from Table 2, the errors of the input impedance and *VSWR* obtained are negligible. Summarized in Table 2 is the results of the training epoch, relative error, and the reflection coefficient for various load impedances at various frequencies.

The conventional GA tuning approach was also carried out for comparison purposes using the same impedance matching network at various frequencies of 1.4 GHz (military and satellite services), 2.3 GHz (LTE networks), 3.4 GHz (radar systems), 4.0 GHz (satellite earth stations), and 5.0 GHz (WiFi band). The numerical results are given in Table 2. It is evident from the table that the proposed AQGA tuning method arrives at the required component value using significantly less iterations in comparison to the CGA tuning method.

The AQGA and CGA tuning algorithms were executed on a Pentium (R) 7 CPU 16 GHz PC. The longest, shortest, and average time for 400 runs for both algorithms are given in Table 3. The proposed AQGA approach obtains the solution much more rapidly compared with the CGA approach.

**Table 3:** Comparison of computation times of AQGA and CGA approaches (unit: seconds).

Frequency band	Algorithm	Shortest	Longest	Average	Compared to CGA the proposed AQGA is faster on average by (%)
1.4 GHz (military & satellite services)	CGA	5.3528	49.3091	26.1073	75
1.4 GHz (military & satellite services)	Proposed AQGA	3.2850	10.8324	6.5049	
2.3 GHz (LTE networks)	CGA	2.9841	52.1520	27.6302	49.2
2.3 GHz (LTE networks)	Proposed AQGA	1.7536	28.9173	14.0371	
3.4 GHz (radar systems)	CGA	6.8453	65.7329	35.2961	64.9
3.4 GHz (radar systems)	Proposed AQGA	4.2740	21.6510	12.3874	
4.0 GHz (satellite earth stations)	CGA	4.9560	42.0845	22.0351	54.7
4.0 GHz (satellite earth stations)	Proposed AQGA	2.0352	17.3842	9.9835	
5.0 GHz (Wi-Fi band)	CGA	3.1271	31.0653	17.9350	52.5
5.0 GHz (Wi-Fi band)	Proposed AQGA	1.0735	15.0252	8.5193	

**Figure 6:**
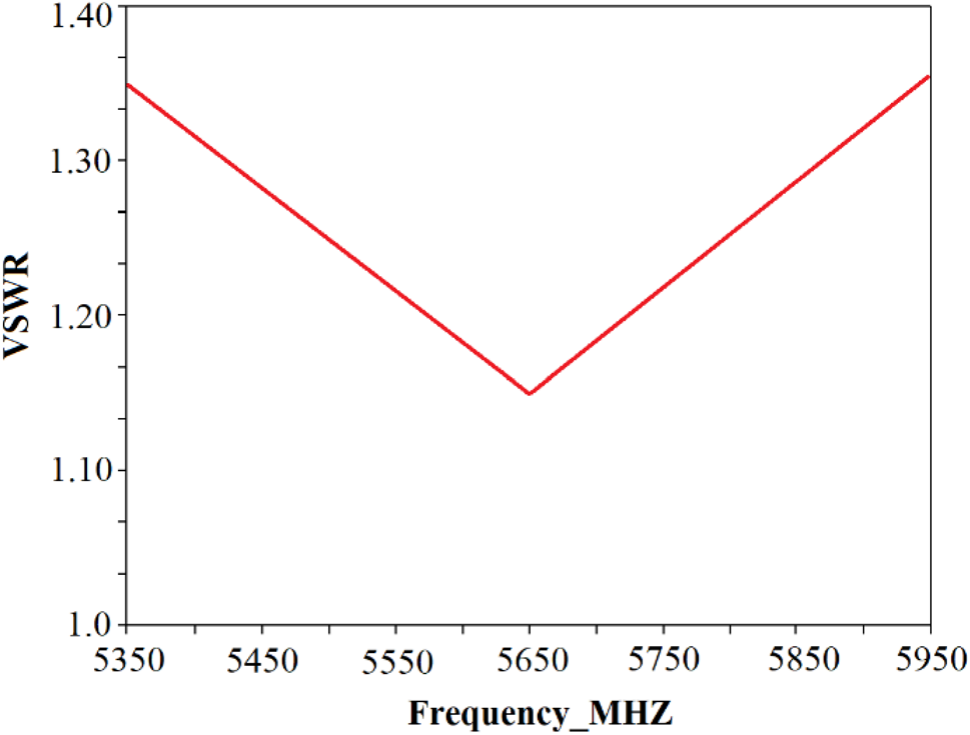
Voltage standing wave ratio (VSWR) over spectrum for WiFi band from 5,350 – 5,925 MHz.

The proposed approach of single frequency tuning can be applied for tuning a given RF band. This involves first tuning the matching network to the center frequency of the operating band of the system. Component values obtained for other frequencies in the given band are then used to compute the VSWR values. As an example, let’s consider the WiFi band (5,350–5,925 MHz) with a bandwidth of 575 MHz. The *T*-type impedance matching network is tuned at the WiFi’s centre frequency of 5.63 GHz for source impedance of $Z_{source}$ = 50 + j30 Ω, load impedance of $Z_{Load}$ = 75 + j50 Ω, and signal amplitude of 1 V. The algorithm provides impedance matching component values of $L_{1}$ = 6.50 nH, $L_{2}$ = 4.36 nH and $C$ = 2.95 *pF*. The VSWR as a function of frequency is shown in Fig. 6 by applying these component values. The VSWR varies in a V-shape response across its band between 1.15 and 1.35. This shows the VSWR performance is acceptable over the whole bandwidth from 5,350 to 5,925 MHz after the tuning algorithm converges.

**Figure 7:**
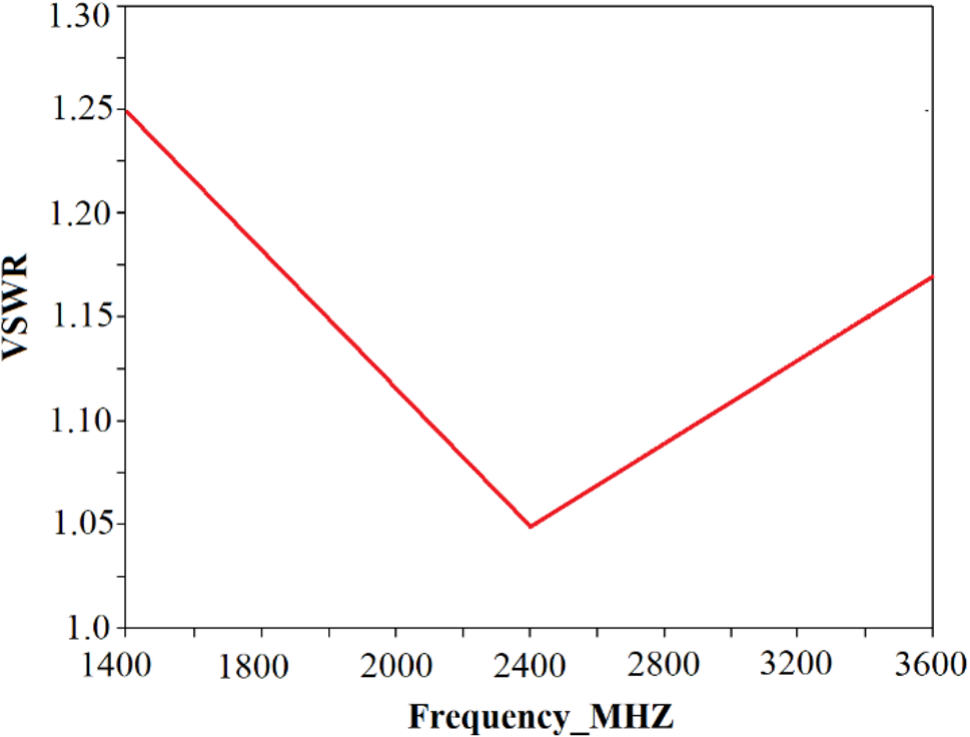
Voltage standing wave ratio (VSWR) over spectrum for ISM band (1,400 – 3,400 MHz).

Figure 7 show the VSWR frequency response for a wider RF band defined between 1,400 and 3,400 MHz, i.e. ISM band in which WLAN, Wi-Fi and Bluetooth operate. In this case the VSWR falls in the range between 1.05 and 1.25. This result shows the effectiveness of the proposed algorithm.

In the case for much wider RF bands, the tuning to the band centre may not give the accuracy required. It is therefore proposed to use multiple frequencies for tuning to realize one set of optimum component values that cover the specified frequency band for a pre-set error.

## Conclusions

Effectiveness of the proposed automatic quantum genetic algorithm (AQGA) is demonstrated against the conventional genetic algorithm (CGA) to adaptively modify the impedance of the matching network to conjugately match the impedance of the antenna with the transmitter’s impedance across the operating frequency band of the wireless system. Optimum matching solution is reached in a significantly shorter time compared to CGA. The proposed algorithm was applied in several scenarios to tune the *T*-type impedance matching *LC*-network at different frequency bands. The results presented verify the effectiveness of the proposed AQGA tuning approach for real-time adaptive antenna tuning.
